# Rapid assessment of malnutrition based on GLIM diagnosis in Crohn’s disease

**DOI:** 10.3389/fnut.2023.1236036

**Published:** 2023-09-06

**Authors:** Longchang Huang, Fu Niannian, Yupeng Zhang, Yifan Shi, Xuejin Gao, Li Zhang, Yan Wu, Cong Dai, Yuhong Huang, Qian Cao, Wei Zhou, Haitao Jiang, Xue Jing, Weiming Zhu, Xinying Wang

**Affiliations:** ^1^Department of General Surgery, Nanjing Jinling Hospital, Affiliated Hospital of Medical School, Nanjing University, Nanjing, China; ^2^Department of Pharmacy, Sir Run Run Shaw Hospital, College of Medicine, Zhejiang University, Hangzhou, China; ^3^Department of Gastroenterology, First Affiliated Hospital, China Medical University, Shenyang, China; ^4^Inflammatory Bowel Disease Center, Sir Run Run Shaw Hospital, College of Medicine, Zhejiang University, Hangzhou, China; ^5^Department of General Surgery, Sir Run Run Shaw Hospital, College of Medicine, Zhejiang University, Hangzhou, China; ^6^Department of Gastrointestinal Surgery, The Affiliated Hospital of Qingdao University, Qingdao, China; ^7^Department of Gastroenterology, The Affiliated Hospital of Qingdao University, Qingdao, China

**Keywords:** HBI, GLIM, Crohn’s disease, malnutrition, nomogram

## Abstract

**Background and aims:**

Malnutrition is strongly linked to adverse outcomes in patients with Crohn’s disease (CD). In this study, our objective was to validate the Global Leadership Initiative on Malnutrition (GLIM) criteria and develop a fast and accurate diagnostic approach for identifying malnutrition in CD patients.

**Methods:**

This study assessed 177 CD patients from four general hospitals. The efficacy of the GLIM criteria for the diagnosis of CD malnutrition was compared. By analyzing the independent factors, a nomogram model was derived and internally validated to predict the diagnosis of malnutrition in patients with CD. Model performance was assessed using discrimination and calibration, decision curves, and net benefit analyses.

**Results:**

Compared with the SGA criteria, the GLIM criteria was consistent in sensitivity (88.89%) and specificity (78.43%) [AUC = 0.84; 95% Confidence Interval (CI): 0.77–0.89]. The Harvey-Bradshaw index(HBI) score (OR: 1.58; 95% CI: 1.15–2.18), body mass index (OR: 0.41; 95% CI: 0.27–0.64), and mid-upper arm circumference (OR: 0.68; 95% CI: 0.47–0.9) were independent factors associated with malnutrition. The nomogram was developed based on these indicators showing good discrimination in malnutrition diagnosis (AUC = 0.953; 95% CI: 0.922–0.984), with agreement after calibration curve and decision curve analysis.

**Conclusion:**

The GLIM criteria are appropriate for diagnosing malnutrition in CD patients. The HBI score may be used to diagnose malnutrition in patients with CD and become a possible selection for the GLIM etiologic criteria of inflammation. The HBM nomogram could be a simple, rapid, and efficient method for diagnosing malnutrition in CD patients.

## 1. Introduction

Crohn’s disease (CD) is emerging as a global health concern due to its increasing prevalence in high-income countries and the anticipated acceleration of cases in developing nations (1). Malnutrition is a frequent complication of Crohn’s disease (CD), primarily caused by reduced oral intake, increased nutrient needs, enhanced gastrointestinal nutrient loss, and, on occasion, drug-nutrient interactions (2). The prevalence of malnutrition in patients with CD varies significantly across different studies, ranging from 12 to 85%. These variations can be attributed to differences in patient characteristics, disease phases, and criteria utilized for diagnosing malnutrition (2, 3). It is widely recognized that malnutrition is linked to higher morbidity and mortality rates, diminished treatment effectiveness, extended hospital stays, and reduced quality of life (QoL) (4, 5), for which nutritional treatment must be administered promptly. Therefore, the European Society for Clinical Nutrition and Metabolism (ESPEN) guidelines recommend that patients with CD should be screened and diagnosed for malnutrition to allow for prompt corresponding nutritional treatment (6). However, there is currently no gold standard for the diagnosis of malnutrition in patients with CD; in addition, there is no clear recommendation from the ESPEN guidelines.

In the past few decades, several tools have been used in the clinical diagnosis of malnutrition in patients with CD. The World Health Organization (WHO) considers the body mass index (BMI) as an essential indicator of malnutrition (7); however, it ignores changes in body composition caused by malnutrition. In 2015, the ESPEN introduced criteria for diagnosing malnutrition, which include body mass index (BMI), unintentional weight loss, and low fat-free mass index (FFMI) (8, 9). It should be noted that the ESPEN criteria, which do not include etiologic factors, may underestimate the prevalence of malnutrition in patients with CD. The Subjective Global Assessment (SGA) is commonly regarded as a “fuzzy, semi-gold” standard for diagnosing malnutrition (10, 11), but it may not be suitable for efficient clinical screening due to its complexity and the rigorous qualifications required for investigators.

In 2018, the GLIM criteria were proposed as the global uniform criteria for the diagnosis of malnutrition (12). The GLIM criteria categorize malnutrition into phenotypic criteria (weight loss, low BMI, and reduced muscle mass) and etiologic criteria (reduced food intake/assimilation and disease burden/inflammation). Individuals who satisfy at least one criterion from each sub-criterion are considered to have malnutrition (13). Its effectiveness in diagnosing malnutrition has been widely demonstrated in many diseases, including elderly community dwellers (14), tumors (15), and cardiovascular diseases (16). However, to date, only five studies have validated its use in Inflammatory bowel disease(IBD) patients (17–21), and two of them are separated patients with CD patients into research (18, 19). Although all studies validated the accuracy of the GLIM criteria for the diagnosis of malnutrition in IBD patients, the etiological criteria used were different. The underlying reason for this phenomenon is that although the GLIM consensus states that disease burden/inflammation is an etiological criterion for malnutrition, it does not address the specific selection of indicators (12, 22).

In addition, In the clinical application of the GLIM criteria, multiple parameters are utilized for diagnosing malnutrition based on the criteria. Due to the complexity and time-consuming nature of using multiple parameters, it would be beneficial to develop a simplified yet accurate clinical approach for diagnosing malnutrition upon admission in patients with CD.

Therefore, the objective of this study was to assess the reliability of the GLIM criteria in diagnosing malnutrition in patients with CD. Furthermore, the study sought to investigate the specific etiological criteria incorporated in the GLIM criteria for diagnosing malnutrition in patients with CD. Moreover, the aim was to develop and validate a simplified, efficient, and precise diagnostic approach for identifying malnutrition in CD patients. Ultimately, this will contribute to the early detection of malnutrition in patients with CD and facilitate timely diagnosis and treatment.

## 2. Materials and methods

### 2.1. Study design and population

This study is a multicenter, prospective, observational study (registration number: ChiCTR2000035720). The study enrolled patients diagnosed with Crohn’s disease at four general hospitals, including the Affiliated Jinling Hospital of Nanjing University, The First Hospital of China Medical University, Sir Run Run Shaw Hospital, and The Affiliated Hospital of Qingdao University, during the period from September 2020 to May 2021. All assessments and data collection were carried out within 48 h of admission for each patient.

**Figure 1:**
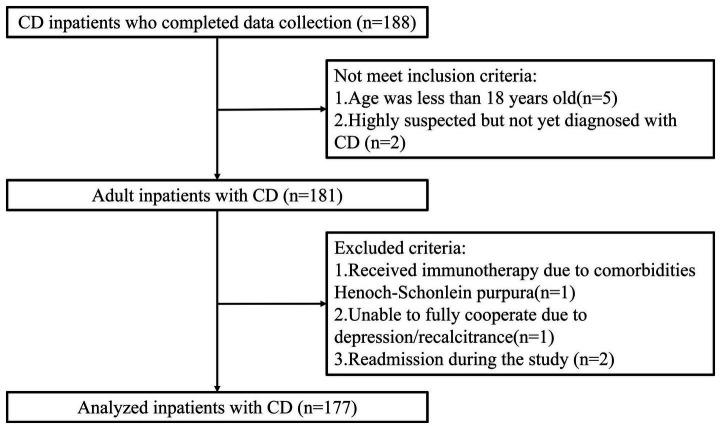
The flow chart of study design and patient selection.

The study’s inclusion criteria were as follows: (1) age ≥ 18 years, (2) diagnosis of Crohn’s disease based on clinical, radiologic, endoscopic, and histologic criteria, and (3) willingness to participate in the study and provide written consent. The exclusion criteria were: (1) emergency surgery for an intestinal fistula or abdominal abscess, (2) unstable vital signs or hemodynamics, (3) pregnant or lactating women, (4) admission due to other critical comorbidities or organ dysfunction, (5) patients with a life expectancy of less than 24 h, (6) immunosuppressive treatment for conditions unrelated to inflammatory bowel disease (such as organ transplantation), (7) contraindications to bioelectrical impedance analysis (BIA), making body composition analysis unsuitable, and (8) patients who had already been selected for participation in other clinical studies (see Figure 1).

### 2.2. Data collection

Within 48 h of admission, researchers collected baseline characteristics including sex, age, height, weight, and history of smoking and drinking. They also gathered information related to inflammatory bowel disease (IBD), recent changes in non-volitional weight loss, and food intake. The Inflammatory Bowel Disease Questionnaire (IBDQ) was used to assess the quality of life (QoL) of patients with Crohn’s disease. The IBDQ is a validated and reliable measurement tool (23). Several malnutrition diagnostic tools, including WHO-related BMI, SGA, ESPEN, and GLIM criteria, were used to assess patients’ nutritional status (12, 17). At each participating center, there were two experienced clinical nutrition physicians and one nutrition specialist nurse involved in the investigation (17).

### 2.3. Measurement of physical performance and body composition

Physical function was evaluated using various measures, including body composition, mid-upper arm circumference (MUAC), muscle strength, the five-repetition sit-to-stand test (5STS), and the 6-meter walk test. The patient’s left arm was in a state of natural sagging when the MUAC was measured. The circumference of the left upper arm was measured at the midpoint twice, and the average value was calculated (24). Other specific measurement methods have been discussed in our previous study (17).

### 2.4. Disease activity

The Crohn’s Disease Activity Index (CDAI) (25) and HBI (26) were used to determine the disease activity of CD. The CDAI score < 150 was defined as disease remission, while CDAI score ≥ 150 was defined as disease activity.

### 2.5. Nomogram for individualized prediction of malnutrition

Multivariate logistic regression analysis was performed using the stepwise selection method based on Akaike’s information criterion to identify independent risk factors for malnutrition in patients with CD. Variables with *p* values < 0.05 were considered as independent predictors. Random forest plots were drawn to demonstrate the precision and importance of the predictors, and incorporated into nomograms for the individualized predictions of malnutrition. To evaluate the performance of the nomogram, the area under the curve (AUC) was calculated to assess discrimination. Additionally, calibration curve analysis was performed with 1,000 bootstrap repetitions to evaluate calibration. Decision curve analysis was conducted to evaluate the clinical utility of the nomogram by calculating the standardized net benefits at different threshold probabilities. Several recommended malnutrition diagnostic tools were set as comparators to the nomogram model, including the WHO-related BMI, ESPEN, and SGA criteria.

### 2.6. Statistical analysis

Categorical variables were presented as frequencies or percentages. For continuous variables, normal distribution was assessed, and the results were reported as mean and standard deviation (SD) if normally distributed, or median and interquartile range (IQR) if not normally distributed. Univariate analysis was performed by Pearson Chi-square test or Fisher exact probability method, and factors with statistical difference (*p* < 0.05) were included in the logistic multivariate regression analysis model (backward selection) to determine independent risk factors for malnutrition. For all analyses, statistical significance was determined using a significance level of *p* < 0.05. The statistical software packages SPSS (version 25.0, IBM Corp., NY, United States) and MedCalc (version 20; MedCalc Software, Ostend, Belgium) were utilized for conducting the statistical analyses.

### 2.7. Ethics statement

The present study received ethical approval from the ethics committee of the Affiliated Jingling Hospital of Nanjing University, as well as the ethics review boards of the three other participating institutions (Ethics approval number: 2019NZKY-026-02). Prior to the commencement of the study, all patients provided informed consent by signing a consent form.

## 3. Results

### 3.1. Baseline characteristics of patients

A total of 177 hospitalized patients with CD (70.1% male and 29.9% female; BMI, 18.9 ± 3. 0 kg/m^2^, mean age, 36.4 ± 13.5 years) were enrolled in the analysis, as shown in Table 1. Using the GLIM criteria, there were 123 patients with CD patients who had malnutrition and 54 patients with CD with a normal nutritional status. Among the malnourished patients, 30 individuals were classified as moderate malnutrition, while 93 individuals were classified as severe malnutrition. There were statistically significant differences between the group of patients with normal nutritional status and the group with malnutrition in the following aspects: body weight, BMI, grip strength (HGS), 5STS, FFMI, MUAC, whole body phase angle (phA), white blood cells (WBC), hemoglobin (HGB), CDAI score, and HBI score (all with *p* < 0.05). The IBDQ score of patients with malnutrition was significantly lower than that of well-nourished patients [155.0 (130.5–177.0) vs. 179.5 (156.8–199.8), *p* < 0.001]. No statistically significant differences were observed between the two groups in terms of age, sex, and duration of disease (*p* > 0.05 for all comparisons).

**Table 1:** Baseline characteristics of 177 CD patients.

		GLIM diagnosis		
	Overall (*n* = 177)	Normal (*n* = 54)	Malnutrition (*n* = 123)	*p*-value
Age, mean ± SD, year	36.4 ± 13.5	37.0 ± 13.4	36.1 ± 13.6	0.698
Sex, No. (%)				0.906
Male	124 (70.1%)	37 (68.5%)	87 (70.7%)	
Female	53 (29.9%)	17 (31.5%)	36 (29.3%)	
Weight, mean ± SD, kg	53.7 ± 10.1	62.1 ± 10.9	50.0 ± 7.2	< 0.001
Height, mean ± SD, cm	168.5 ± 8.6	167.9 ± 7.1	168.8 ± 8.4	0.509
BMI, mean ± SD, kg/m^2^	18.9 ± 3.0	21.9 ± 2.6	17.5 ± 2.0	< 0.001
Disease duration, median (IQR), month	24 (12–84)	24 (12–75)	24 (12–96)	0.481
NRS2002, No. (%)				< 0.001
<3	38 (21.5%)	38 (70.4%)	0 (0%)	
≥3	139 (78.5%)	16 (29.6%)	123	
HGS, median (IQR), kg	30.4 (23.6–38.0)	34.9 (26.2–41.8)	29.5 (22.1–36.5)	0.001
6-m walk time, median (IQR), m/s	1.20 (1.10–1.40)	1.21 (1.14–1.42)	1.23 (1.12–1.38)	0.734
5STS, median (IQR), s	9.3 (7.1–11.2)	8.2 (5.6–10.4)	9.6 (7.6–11.4)	0.007
FFMI, median (IQR), kg/m^2^	14.8 (13.4–16.3)	16.9 (15.0–18.1)	14.2 (13.2–15.5)	< 0.001
MUAC, median (IQR), cm	24.2 (22.0–26.0)	26.7 (25.4–28.7)	22.8 (21.4–24.6)	< 0.001
PhA, median (IQR)	5.3 (4.7–5.9)	5.5 (4.9–6.1)	5.2 (4.7–5.8)	0.027
HGB, median (IQR), g/L	121 (107–137)	128 (118–143)	116 (106–134)	0.002
Serum albumin,median (IQR), g/L	39.4 (36.2–43.1)	40.3 (38.5–43.1)	39.1 (35.9–43.1)	0.162
WBC, median (IQR), x10ˆ9/L	4.7 (3.7–6.7)	5.42 (4.2–7.1)	4.5 (3.5–6.2)	0.022
NLR, median (IQR)	2.2 (1.7–3.4)	2.04 (1.6–2.8)	2.36 (1.7–3.7)	0.069
CRP, median (IQR), mg/L	2.9 (0.5–16.8)	3.3 (0.7–8.8)	2.6 (0.5–16.9)	0.592
CDAI score, median (IQR)	156.2 (116.1–200.4)	117.6 (91.1–165.2)	168.5 (133.8–216.0)	< 0.001
HBI score, median (IQR)	5.0(3.5–7.0)	4.0 (2.0–5.0)	6.0 (4.0–7.0)	< 0.001
Disease behavior of CD, No. (%)				< 0.001
B1	60 (33.9%)	30 (50%)	30 (50%)	
B2	84 (47.5%)	14 (16.7%)	70 (83.3%)	
B3	33 (18.6%)	10 (30.3%)	23 (69.7%)	
Disease location of CD, No. (%)				0.41
L1	76 (42.9%)	20 (26.3%)	56 (73.7%)	
L2	21 (11.9%)	9 (40.9%)	13 (59.1%)	
L3	33 (18.6%)	25 (31.3%)	55 (68.8%)	
Smoking history, No. (%)				0.629
NO	142 (80.2%)	45 (83.3%)	97 (78.9%)	
Yes	35 (19.8%)	9 (16.7%)	26 (21.1%)	
Alcohol consumption, No. (%)				0.259
NO	161 (91%)	47 (87.0%)	114 (92.7%)	
Yes	16 (9.0%)	7 (13.0%)	9 (7.3%)	
Surgery history, No. (%)				0.374
NO	78 (44.1%)	27 (15.3%)	51 (28.8%)	
Yes	99 (55.9%)	27 (15.3%)	72 (40.7%)	
GLIM malnutrition grading, No. (%)				——
Stage1	——	0 (0%)	30 (24.4%)	
Stage2	——	0 (0%)	93 (75.6%)	
Total IBDQ score, median (IQR)	161.0 (139.5–182)	179.5 (156.8–199.8)	155.0 (130.5–177.0)	< 0.001
Bowel symptoms, median (IQR)	56.0 (48.0–64.0)	62.0 (54.3–66.8)	53.0 (46.5–61.5)	< 0.001
Systemic symptoms, median (IQR)	25.0 (19.0–29.0)	27.0 (25.0–31.0)	24.0 (18.5–27.5)	< 0.001
Emotional function, median (IQR)	60.0 (50.0–69.0)	65.5(54.8–72.3)	56(49.0–67.0)	< 0.001
Social function, median (IQR)	21.0 (15.0–29.0)	28.5 (19.3–32.8)	19.0 (14.0–26.0)	< 0.001

*p* value, Normal group vs. malnutrition group. SD, standard deviation; IQR, interquartile range; GLIM, Global Leadership Initiative on Malnutrition; BMI, body mass index;CD, Crohn’s disease;HGS, Handgrip strength; 5STS, five-repetition sit-to-stand test; FFMI, fat-free mass index; PhA, phase angle; HGB: hemoglobin; WBC, white blood cell;CRP, C-reactive protein; NLR, neutrophil-to-lymphocyte ratio;CDAI, crohn’s disease activity index;HBI, Harvey Bradshaw Index;IBDQ,Inflammatory Bowel Disease Questionnaire. The BMI and FFMI were calculated as Weight (kg) and FFM (kg) divided by squared height (m), respectively.

### 3.2. Concordance of GLIM criteria

**Figure 2:**
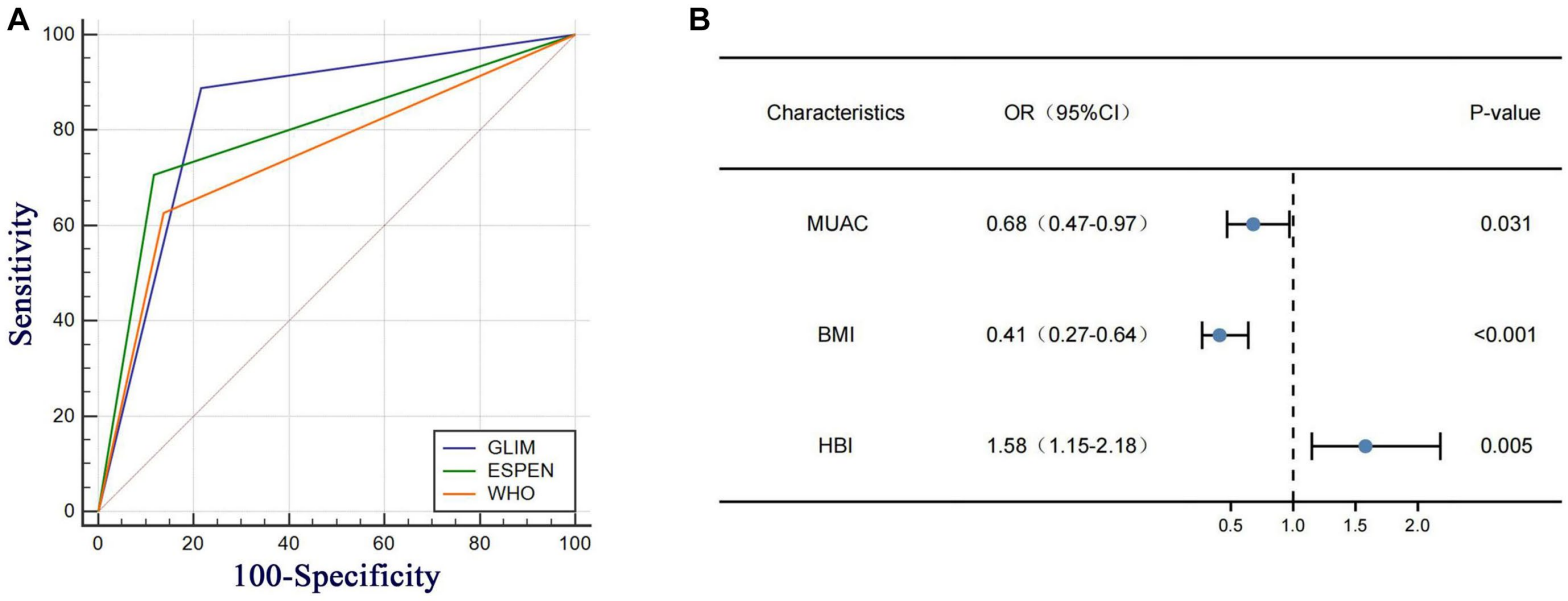
**(A)** Ana under the ROC curves (ALC) for the diagnosis of malnutrition in CD patients. **(B)** Multivariate Cox regression analysis based on GLIM domains and clinical variables in CD patients at rok of malnutrition HBI, Harvey Bradshaw Index Score, MUAC mad-upper arm circumference BMI body mass index.

Table 2 demonstrates that the GLIM criteria exhibited a significant and robust agreement (*K* = 0.662, *p* < 0.001) with the SGA criteria. When compared to the SGA criteria, the ESPEN criteria were superior to the WHO criteria in all aspects of data on malnutrition diagnosis in patients with CD，while the GLIM criteria were superior to the ESPEN criteria in sensitivity (88.89% vs. 70.64%) and negative predictive value (78.43% vs. 54.88%), and the specificity (78.43% vs. 88.24%) and positive predictive value (91.06% vs. 93.68%) were not far behind those of the ESPEN criteria. According to the ROC curve analysis results (Figure 2A; Table 2), the area under ROC curve (AUC) of the GLIM criteria was AUC = 0.84 (95% CI, 0.77–0.89), whereas the WHO criteria (AUC = 0.75, 95 %CI 0.73–0.85) and ESPEN criteria (AUC = 0.79, 95% CI 0.73–0.85) had a lower AUC than the GLIM criteria.

**Table 2:** Accuracy, Sensitivity, Specificity, NPV, PPV, Kappa and AUC for different malnutrition diagnostic tools.

	GLIM-SGA	WHO-SGA	ESPEN-SGA
Sensitivity (%)	88.89 (82.06–93.79)	62.70 (53.64–71.15)	70.64 (61.86–78.41)
Specificity (%)	78.43 (64.68–88.71)	86.28 (73.75–94.30)	88.24 (76.13–95.56)
AUC	0.84 (0.77–0.89)	0.75 (0.67–0.81)	0.79 (0.73–0.85)
PPV (%)	91.06 (85.74–94.52)	91.86 (84.84–95.79)	93.68 (87.40–96.94)
NPV (%)	74.07 (63.07–82.70)	48.35 (42.13–54.62)	54.88 (47.68–61.88)
kappa	0.662	0.397	0.499
*p*-value	<0.001	<0.001	<0.001

*K* value derived from Cohen kappa statistics, data are expressed as Percentage or Kappa value and 95% CI; The latter of each column titles serves as reference criterion in statistic. WHO, World Health Organization; SGA, Subjective Global Assessment; ESPEN, European Society for Clinical Nutrition and Metabolism; GLIM, Global Leadership Initiative on Malnutrition; PPV, positive predictive values; NPV, negative predictive values; AUC, area under the curve.

### 3.3. Independent predictors of malnutrition

Logistic univariate and multiple regression analyses were performed for the two groups of patients with CD diagnosed using the GLIM criteria (Table 3). After univariate logistic regression analysis, BMI, HGS, 5STS, FFMI, MUAC, phA, WBC, HGB, CDAI score, and HBI score were found to be independent factors related to malnutrition in patients with CD (all *p* < 0.05). These variables were included in the multivariate logistic regression analysis. After stepwise variable screening, three variables were screened as independent factors related to malnutrition in CD patients (all *p* < 0.05), including BMI (OR: 0.41; 95% CI: 0.27–0.64), MUAC (OR: 0.68; 95% CI: 0.47–0.97), and HBI score (OR: 1.58; 95% CI: 1.15–2.18). A random forest plot was constructed to show the precision and importance of these predictors (Figure 2B). We also conducted stratified analysis based on CDAI scores for patients in remission and those with active Crohn’s disease, and obtained similar results (Supplementary Table S1).

**Table 3:** Univariable and multivariable logistic regression analyses on factors associated with malnutrition.

	Univariable analyses		Multivariable analyses	
	OR (95%CI)	*p*-value	OR (95%CI)	*p*-value
BMI， mean ± SD， kg/m^2^	0.37 (0.27–0.50)	< 0.001	0.41 (0.27–0.64)	<0.001
HGS， median (IQR)， kg	0.94 (0.91–0.98)	0.002	—	—
5STS， median (IQR) ，s	1.19 (1.05–1.35)	0.009	—	—
FFMI， median (IQR)， kg/m^2^	0.51 (0.40–0.64)	< 0.001	—	—
MUAC， median (IQR)， cm	0.45 (0.35–0.58)	< 0.001	0.68 (0.47–0.97)	0.031
PhA， median (IQR)	0.67 (0.47–0.96)	0.029	—	—
WBC， median (IQR)， x10ˆ9/L	0.85 (0.73–0.99)	0.035	—	—
HGB， median (IQR)， g/L	0.98 (0.96–0.99)	0.005	—	—
CDAI score， median (IQR)	1.01 (1.01–1.02)	< 0.001	—	—
HBI score， median (IQR)	1.62 (1.33–1.98)	< 0.001	1.58 (1.15–2.18)	0.005

*p* value, Normal group vs. malnutrition group. CI, Confidence interval;SD, standard deviation; IQR, interquartile range; BMI, body mass index; 5STS, five-repetition sit-to-stand test; FFMI, fat-free mass index; PhA, phase angle; HGB: hemoglobin; WBC, white blood cell; CDAI, crohn’s disease activity index; HBI, Harvey Bradshaw Index.

### 3.4. Development and evaluation of the HBM nomogram

**Figure 3:**
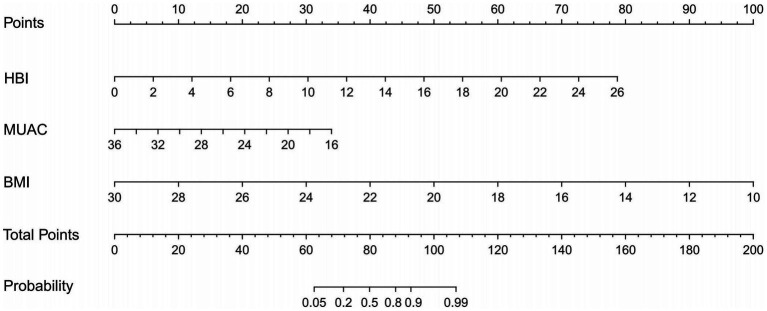
The HBM nomogram is intended for the diagnosis of malnutrition in CD patients (To use the nomogram, an individual patient’s value is located on each variable axis, and a line is drawn upward to determine the number of points received for each variable’s value The sum of these numbers is located on the Total Points axis, and a line is drawn downward to the Probability axes to determine the likelihood of malnutrition). HBI, Harvey Bradshaw Index Score; MUAC, mid-upper arm circumference; BMI, body mass index.

**Figure 4:**
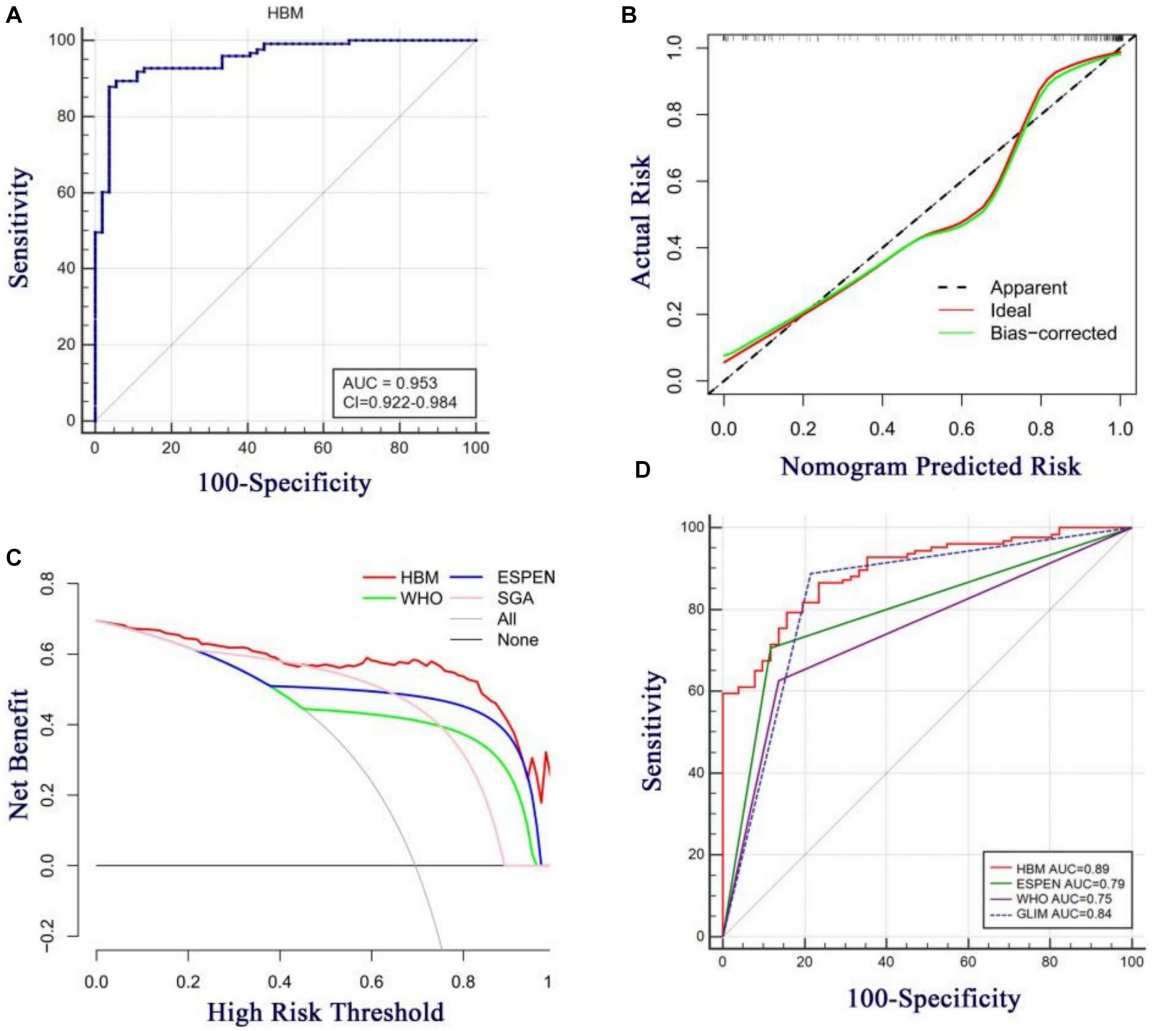
**(A)** Area under the ROC curves (AUC) for the diagnosis of malnutrition using HBM nomogram in CD patients. **(B)** The calibration curve for the risk of malnutrition in CD patients. The nomogram-predicted probability of malnutrition is plotted on the x-axis the actualrisk of malnutrition is plotted on the y-axis. **(C)** Decision curve analysis of the HBM nomogram (red line) compared with different diagnostic criterias. The gray line denotes the assumption that all patients have malnutrition. The thick black line represents the assumption that no patients have malnutrition. **(D)** Area under the ROC curves (AUC) for the diagnosis of malnutrition using different diagnostic criterias in CD patients.

As shown in Figure 3, the three identified independent predictors (BMI, MUAC, and HBI scores) were incorporated into the HBM nomogram to predict malnutrition in patients with CD. Taking the study samples as the training group, 1,000 sampling times were repeated with five-fold cross-validation for internal validation, and the C-index was calculated to be 0.945. Subsequently, a receiver operating characteristic curve (ROC) was plotted, and the area under the ROC curve (AUC) was calculated to evaluate the diagnostic performance of the HBM nomogram. To examine the consistency of the model, a calibration curve was constructed. As shown in Figure 4A, the AUC value of the HBM nomogram was 0.953 with a 95% CI of 0.922–0.984. Figure 4B demonstrates that the nomogram calibration curve for malnutrition probability displayed favorable agreement between the predicted probabilities and actual observations. In summary, the HBM nomogram exhibited strong discriminatory ability and reliable calibration performance for the diagnosis of malnutrition in CD patients.

To assess the clinical utility of the HBM nomogram, a decision curve analysis (DCA) was conducted as indicated in Figure 4C. According to the decision curve, when the threshold probability for a specific patient is above 0, utilizing the nomogram to predict malnutrition provides greater net benefit compared to either treating all patients or employing no treatment strategy. Moreover, the HBM nomogram demonstrated superior benefits across all threshold probability values in comparison to the three diagnostic criteria (the WHO, ESPEN, and SGA criteria), indicating that under the premise of using the GLIM criteria as the diagnostic gold standard, the HBM nomogram may create better clinical effects than the WHO, ESPEN, or SGA criteria in the diagnosis of malnutrition in patients with CD. As shown in Figure 4D, with the SGA diagnosis as the gold standard, the HBM nomogram model also has high diagnostic efficacy compared with the GLIM, ESPEN, and WHO criteria; in addition, it is even slightly better than GLIM diagnosis [(AUC = 0.89, 95% CI: 0.84–0.94) vs. (AUC = 0.84, 95% CI: 0.77–0.89)]. These results indicate that the HBM nomogram has promising clinical applications.

## 4. Discussion

In the present study, we investigated the validity of the GLIM criteria for the diagnosis of malnutrition in patients with CD and observed higher HBI scores in patients with malnutrition compared with those with normal nutritional status. For the first time, we used the HBI score as an independent diagnostic parameter for identifying malnutrition in patients with CD. Additionally, we integrated BMI and MUAC to develop and validate a simple, efficient, and precise diagnostic method for malnutrition in CD patients.

Our study found that the prevalence of malnutrition in CD patients was 69.5%, according to the GLIM criteria, in line with previous findings of studies that employed the GLIM criteria as a standard (18, 19, 21). Two studies that used the GLIM criteria showed that malnutrition prevalence in patients with CD was 34% (20) and 33% (27). Considering that the two studies were from the same hospital as a single-center study, the small sample size may be primarily responsible for the low prevalence of malnutrition in the studies. Despite the high incidence of malnutrition diagnosis according to the GLIM criteria, it appears to be a suitable approach for accurately identifying malnutrition in patients. Camilla et al. suggested that the GLIM criteria had a higher rate of malnutrition detection than ESPEN 2015 (20), which is consistent with our findings (Figure 2A). In addition, studies by Fiorindi et al. (27) and Li et al. (18) have proposed that the GLIM criteria may be more suitable for evaluating the nutritional status of patients with CD compared to other tools like NRS-2002, MUST, MST, and MIRT. Our results showed that the GLIM criteria indeed had better concordance with the SGA criteria than the ESPEN and WHO criteria, especially in sensitivity (88.89% vs. 70.64% vs. 62.70%) and negative predictive value (78.43% vs. 54.88% vs. 42.35%). To date, all studies have suggested that the GLIM criteria serve as an appropriate tool for the diagnosis of malnutrition in CD.

Owing to the use of the GLIM criteria, current research has gradually recognized the important role of etiological criteria for the diagnosis of malnutrition; however, they still focus more on the phenotypic criteria, ignoring the specific selection of the etiological criteria for the GLIM criteria, resulting in diversity. In three studies investigating inflammatory bowel disease (IBD), the plasma C-reactive protein (CRP) level was examined as a specific marker for the GLIM etiological criteria of inflammation (17, 18, 21), wheras other studies only provide a broad definition (19, 20).

However, Huang et al. (21) showed that there was no significant difference in CRP levels between the malnutrition and normal groups in IBD patients (21). In two studies of Crohn’s disease, CRP levels were significant between the two groups of patients, as defined by the GLIM criteria, but neither was an independent factor for malnutrition (18, 19). Furthermore, our results showed that there was no significant difference in the biochemical markers of inflammation (CRP, WBC, and NLR) between the malnutrition and normal groups in patients with CD. This indicates that further research should be conducted to explore whether CRP can be used as a specific measure of the GLIM etiological criteria for inflammation.

During the analysis of the inflammatory etiology criteria for the GLIM criteria, our results showed that HBI score was independently associated with GLIM-defined malnutrition (OR: 1.58; 95% CI: 1.15–2.18). HBI is associated with nutrient deficiencies in malnourished patients, which further supports our results (28, 29). In 1980, Harvey and Bradshaw proposed using the HBI score instead of the CDAI to assess CD disease activity (30). Compared with the CADI, the HBI score is more concise and has been widely used in the assessment of disease activity in patients with CD (26, 31). Taking into account that the HBI score consists of five components: abdominal pain, abdominal mass, general well-being,number of liquid stools per day, and complication (26), it appropriately covers the current GLIM criteria for inflammatory etiology diagnosis (12), showing the its significant application prospects in the GLIM criteria. However, further research is required as no existing study has utilized the HBI score as an independent predictor for diagnosing malnutrition in CD patients.

To simplify the diagnostic process, we developed the HBM nomogram model. Although there are many nomogram models for the prediction of postoperative complications in CD (32, 33), no nomogram model for the prediction of dystrophy in CD has been developed and evaluated. Our HBM nomogram model was not only the first nomogram developed for diagnosing malnutrition in CD patients; it also showed high performance in diagnosing malnutrition. Using GLIM criteria as the standard, its AUC for discriminating patients with CD-associated malnutrition was 0.953 (0.922–0.984), and if the SGA criteria was used as the gold standard for diagnosis, the HBM nomogram was slightly better than the GLIM criteria in distinguishing malnutrition (AUC: 0.89 vs. 0.84). High diagnostic efficacy was associated with the incorporation of BMI changes, measurement of muscle mass loss (MUAC), and etiological criteria (HBI score). In addition, this is the first study to show that the HBI score is a diagnostic indicator of malnutrition.

Significantly, compared to other forecasting models (34–36), the HBM nomogram is simpler to use and can quantify metrics without the need for additional tools or calculations. The three predictor metrics involved in the HBM nomogram model can be obtained by simple questioning and anthropometric measurements on patients, which will greatly improve the efficiency of outpatient doctors in assessing whether patients with CD patients malnourished and help guide subsequent nutritional treatment. Furthermore, the HBM nomogram will play an important role in developing and remote regions or countries without laboratory testing and technical conditions, since the model is non-invasive and cost-effective, compared to current advanced anthropometric techniques, such as blood tests, dual energy absorptiometry, bioelectrical impedance, ultrasound, computed tomography, and magnetic MRI.

We acknowledge some limitations in the study. First, our nomogram model can only diagnose malnutrition in patients with CD and cannot distinguish the severity of malnutrition. Second, there is a lack of external validation cohorts due to the limitation of the sample size; therefore, it is necessary to further expand the sample size and conduct further research to improve the evaluation model. Nevertheless, we are cautiously optimistic that the HBM nomogram may be useful once externally validated.

## 5. Conclusion

In summary, the GLIM criteria are appropriate for diagnosing malnutrition in patients with CD. In addition, we developed and validated a simple, rapid, and efficient nomogram model for malnutrition diagnosis in patients with CD, and external validation in independent cohorts is recommended. This is the first study to use the HBI score as a diagnostic indicator of malnutrition, thus suggesting that the HBI score may be a possible selection for the GLIM etiological criteria of inflammation.

## Data availability statement

The original contributions presented in the study are included in the article/Supplementary material, further inquiries can be directed to the corresponding authors.

## Ethics statement

The studies involving human participants were reviewed and approved by The Affiliated Jingling Hospital of Nanjing University (Ethics approval number: 2019NZKY-026-02). The patients/participants provided their written informed consent to participate in this study.

## Author contributions

The study was conceived and designed by XW and WZhu. The trial protocol was designed and drafted by XW, YZ, and LZ. The statistical analysis plan was contributed by LH and YS, who also performed the statistical analysis. LH, FN, and YS were involved in drafting and critically revising the manuscript. The research was conducted by YZ, CD, YH, YW, WZho, QC, XJ, and HJ, who were responsible for the data collection and hands-on work. Data curation and quality checking were carried out by LH, YZ, CD, YW, and XJ. All authors critically revised and approved the final version of the manuscript, and share responsibility for its content.

## Funding

This work was funded by the 13th Five-Year Plan Foundation of Jiangsu Province for Medical Key Talents (ZDRCA2016091) and the Science Foundation of Outstanding Youth in Jiangsu Province (BK20170009).

## Conflict of interest

The authors declare that the research was conducted in the absence of any commercial or financial relationships that could be construed as a potential conflict of interest.

## Publisher’s note

All claims expressed in this article are solely those of the authors and do not necessarily represent those of their affiliated organizations, or those of the publisher, the editors and the reviewers. Any product that may be evaluated in this article, or claim that may be made by its manufacturer, is not guaranteed or endorsed by the publisher.

## Supplementary material

The Supplementary material for this article can be found online at: https://www.frontiersin.org/articles/10.3389/fnut.2023.1236036/full#supplementary-material
