# Supplementary material for: Rapid assessment of malnutrition based on GLIM diagnosis in Crohn’s disease
Source: Front Nutr. 2023 Sep 6;10:1236036. doi: 10.3389/fnut.2023.1236036 (PMC10510406; doi:10.3389/fnut.2023.1236036)
Supplement: Supplementary file 1 [file Table_1.docx]

| **Supplementary Table 1** Univariable and Multivariable Logistic Regression Analyses on Factors Associated With Malnutrition after stratification of CDAI | | | | | | | | | | | |
| --- | --- | --- | --- | --- | --- | --- | --- | --- | --- | --- | --- |
|  | CDAI<150(n=79) | | | | |  | CDAI≥150(n=98) | | | | |
|  | Univariable analyses | |  | Multivariable analyses | |  | Univariable analyses | |  | Multivariable analyses | |
|  | OR（95%CI） | P-value |  | OR（95%CI） | P-value |  | OR（95%CI） | P-value |  | OR（95%CI） | P-value |
| BMI，mean±SD，kg/m² | 0.36(0.23 - 0.58) | < 0.001 |  | 0.24 (0.09 - 0.62) | 0.003 |  | 0.35(0.21 - 0.58) | < 0.001 |  | 0.43(0.22 - 0.84) | 0.014 |
| HGS，median (IQR)，kg | 0.94(0.89 - 0.99) | 0.022 |  | — | — |  | 0.94 (0.88 - 0.99) | 0.044 |  | — | — |
| 5STS，median (IQR)，s | 1.24 (1.02 - 1.50) | 0.034 |  | — | — |  | 1.12 (0.92 - 1.36) | 0.270 |  | — | — |
| FFMI，median (IQR)，kg/m² | 0.60(0.46 - 0.79) | < 0.001 |  | — | — |  | 0.38(0.24 - 0.61) | < 0.001 |  | — | — |
| MUAC，median (IQR)，cm | 0.45 (0.32 - 0.64) | < 0.001 |  | 0.68（0.47-0.97） | 0.031 |  | 0.37 (0.23 - 0.60) | < 0.001 |  | 0.38 (0.23 - 0.61) | < 0.001 |
| PhA，median (IQR) | 0.97 (0.57 - 1.63) | 0.896 |  | — | — |  | 0.82(0.44 - 1.53) | 0.540 |  | — | — |
| WBC，median (IQR)，x10ˆ9/L | 0.73 (0.56 - 0.95) | 0.021 |  | 0.49(0.25 - 0.95) | 0.035 |  | 0.85(0.67 - 1.08) | 0.182 |  | — | — |
| HGB，median (IQR)，g/L | 0.97 (0.94 - 1.01) | 0.091 |  | — | — |  | 1.00 (0.98 - 1.03) | 0.761 |  | — | — |
| CDAI score，median (IQR) | 1.02 (1.01 - 1.04) | 0.008 |  | — | — |  | 1.00(0.99 - 1.01) | 0.596 |  | — | — |
| HBI score，median (IQR) | 1.78 (1.28 - 2.48) | < 0.001 |  | 2.90(1.27 - 6.64) | 0.012 |  | 1.33 (1.02 - 1.73) | 0.034 |  | 1.30 (1.01 - 1.68) | 0.049 |

P value, Normal group vs. malnutrition group.

CI, Confidence interval;SD, standard deviation; IQR, interquartile range; BMI, body mass index; 5STS, five-repetition sit-to-stand test; FFMI, fat-free mass index; PhA, phase angle; HGB: hemoglobin; WBC, white blood cell;CDAI, crohn’s disease activity index;HBI, Harvey Bradshaw Index.
